# Lipopolysaccharide-Induced Matrix Metalloproteinase-9 Expression Associated with Cell Migration in Rat Brain Astrocytes

**DOI:** 10.3390/ijms21010259

**Published:** 2019-12-30

**Authors:** Chien-Chung Yang, Chih-Chung Lin, Li-Der Hsiao, Jing-Ming Kuo, Hui-Ching Tseng, Chuen-Mao Yang

**Affiliations:** 1 Department of Traditional Chinese Medicine, Chang Gung Memorial Hospital at Tao-Yuan, Kwei-San, Tao-Yuan 33302, Taiwan; r55161@cgmh.org.tw; 2 School of Traditional Chinese Medicine, College of Medicine, Chang Gung University, Kwei-San, Tao-Yuan 33302, Taiwan; 3 Department of Anesthetics, Chang Gung Memorial Hospital at Linkuo, Kwei-San, Tao-Yuan 33302, Taiwan; chihchung@adm.cgmh.org.tw; 4 Department of Pharmacology, College of Medicine, China Medical University, Taichung 40402, Taiwan; lidesiao@livemail.tw (L.-D.H.); roro31009@gmail.com (J.-M.K.); huiching1205@yahoo.com.tw (H.-C.T.); 5 Department of Post-Baccalaureate Veterinary Medicine, College of Medical and Health Science, Asia University, Wufeng, Taichung 41354, Taiwan

**Keywords:** neuroinflammation, neurodegeneration, astrocytes, LPS, MMP-9

## Abstract

Neuroinflammation is a landmark of neuroinflammatory and neurodegenerative diseases. Matrix metalloproteinase (MMP)-9, one member of MMPs, has been shown to contribute to the pathology of these brain diseases. Several experimental models have demonstrated that lipopolysaccharide (LPS) exerts a pathological role through Toll-like receptors (TLRs) in neuroinflammation and neurodegeneration. However, the mechanisms underlying LPS-induced MMP-9 expression in rat brain astrocytes (RBA-1) are not completely understood. Here, we applied pharmacological inhibitors and siRNA transfection to assess the levels of MMP-9 protein, mRNA, and promoter activity, as well as protein kinase phosphorylation in RBA-1 cells triggered by LPS. We found that LPS-induced expression of pro-form MMP-9 and cell migration were mediated through TLR4, proto-oncogene tyrosine-protein kinase (c-Src), proline-rich tyrosine kinase 2 (Pyk2), platelet-derived growth factor receptor (PDGFR), phosphoinositide 3-kinase (PI3K)/protein kinase B (Akt), p38 mitogen-activated protein kinase (MAPK), and Jun amino-terminal kinase (JNK)1/2 signaling molecules in RBA-1 cells. In addition, LPS-stimulated binding of c-Jun to the MMP-9 promoter was confirmed by chromatin immunoprecipitation (ChIP) assay, which was blocked by pretreatment with c-Src inhibitor II, PF431396, AG1296, LY294002, Akt inhibitor VIII, p38 MAP kinase inhibitor VIII, SP600125, and tanshinone IIA. These results suggest that in RBA-1 cells, LPS activates a TLR4/c-Src/Pyk2/PDGFR/PI3K/Akt/p38 MAPK and JNK1/2 pathway, which in turn triggers activator protein 1 (AP-1) activation and ultimately induces MMP-9 expression and cell migration.

## 1. Introduction

Astrocytes are specialized glial cells that constitute nearly half of the number of brain cells. They play many important roles, such as nourishing neurons, metabolism of neurons, and regulation of concentrations of ions, neurotransmitters, and pH in the extracellular space in the central nerve system. They also have roles in the modulation of synaptic signaling and are components of the blood–brain barrier (BBB) [1]. Moreover, astrocytes can promote the survival of neurons and other glia by releasing neurotrophic and gliotrophic factors [2]. However, in contrast, their character is also like immune cells. Serval lines of evidence have shown that astrocytes can release inflammatory mediators such as tumor necrosis factor-alpha (TNFα), interleukin-1 beta (IL-1β), and matrix metalloproteinase-9 (MMP-9) [3] to participate in pathological processes of brain insults, such as Alzheimer′s disease and other age-associated dementias [4], multiple sclerosis [5], major depressive disorder [6], and Parkinson′s disease [7]. Thus, astrocytes play an important role in several neuroinflammatory and neurodegenerative processes.

MMPs are a series of zinc-dependent endopeptidases that consist of more than twenty MMPs in human and other species. Their core functions are capable of degrading extracellular matrix (ECM) proteins as well as cytokines, chemokines, growth factors, other MMPs, cell surface receptors, and serine proteinase inhibitors. They are typically either secreted or anchored to the cell surface. MMPs, especially MMP-9, play several physiological and pathological roles in brain development and disorders [8]. The up-regulation of MMP-9 is associated with BBB breakdown, demyelination, inflammation, and neurotoxicities in many central nervous system (CNS) diseases [8,9,10]. Several reports have demonstrated that MMP-9 could be induced by a series of stimuli, including lipopolysaccharide (LPS) in the brain, including astrocytes [11,12]. Actually, abnormal expression and activation of MMP-9 induced by infection leads to the breakdown of the ECM, the disruption of the BBB; preventing normal cell signaling; and, eventually, cell death during the inflammatory responses [8,13].

The contribution of MMP-9 in CNS infection, including bacterial and viral meningitis, has been found for decades [14]. These links are made by the finding of BBB damage by MMP-9 in bacterial meningitis [12]. Furthermore, inhibition of MMPs with the GM6001 combined with ceftriaxone has beneficial effects, including attenuated neuronal injury and improved learning, as shown on a rat model of *Streptococcus pneumoniae* meningitis [15]. LPS, one of the Gram-negative bacterial components, is known as a potent pathogenesis of bacterial endotoxin. LPS usually induces immune and inflammatory responses through toll-like receptor 4 (TLR4) and downstream signaling components [16,17]. Previous studies have shown that LPS can activate several downstream signaling molecules of TLR4, such as proto-oncogene tyrosine-protein kinase (c-Src) [18], proline-rich tyrosine kinase 2 (Pyk2) [19], platelet-derived growth factor receptor (PDGFR)/phosphoinositide 3-kinase (PI3K)/protein kinase B (Akt) [18], and mitogen-activated protein kinases (MAPKs) [20] to trigger activator protein 1 (AP-1) activity [17] and enhance the expression of inflammatory proteins, including MMP-9, monocyte chemotactic protein-1, IL-8, and intercellular adhesion molecule-1 (ICAM-1), in various types of cells. LPS can also induce MMP-9 expression in macrophages and animals [20,21]. However, in rat brain astrocytes (RBA-1) cells, the detailed mechanisms underlying MMP-9 expression induced by LPS is not well understood.

In the present study, we dissected the signaling component-linked AP-1 activation to MMP-9 expression induced by LPS in RBA-1 cells. Our results demonstrated that LPS-induced MMP-9 expression was mediated through TLR4/c-Src/Pyk2/PDGFR/PI3K/Akt/p38 MAPK and Jun amino-terminal kinase (JNK)1/2-dependent activation of AP-1 associated with cell migration in RBA-1 cells.

## 2. Results

### 2.1. LPS Induced MMP-9 Expression through Transcription and Translation

**Figure 1:**
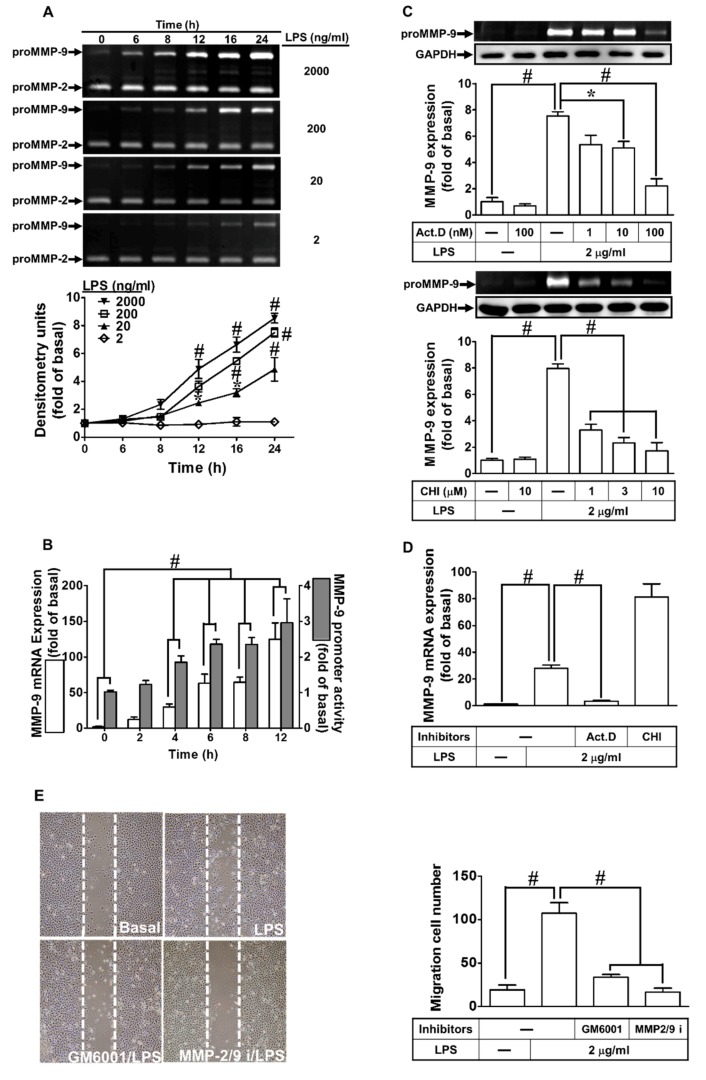
Lipopolysaccharide (LPS) induced metalloproteinase-9 (MMP-9) expression and cell migration in rat brain astrocytes (RBA-1) cells. (**A**) Cells were incubated with various concentrations of LPS (2, 0.2, 0.02, and 0.002 μg/mL) for the indicated time intervals (0, 6, 8, 12, 16, and 24 h). The levels of MMP-9 were determined by gelatin zymography. (**B**) Cells were incubated with LPS (2 μg/mL) for the indicated time intervals (0, 2, 4, 6, 8, and 12 h). The levels of MMP-9 mRNA and promoter activity were analyzed by real-time PCR and promoter activity assay, respectively. (**C**) Cells were pretreated with actinomycin D (Act. D; 0.001, 0.01, and 0.1 μM) or cycloheximide (CHI; 1, 3, and 10 μM) for 1 h, and then incubated with LPS (2 μg/mL) for 24 h. The levels of MMP-9 were determined by gelatin zymography. The glyceraldehyde-3-phosphate dehydrogenase (GAPDH) level of cell lysates was assayed by western blot. (**D**) Cells were pretreated with Act. D (0.1 μM) or CHI (10 μM) for 1 h, and then incubated with LPS (2 μg/mL) for 4 h. The level of MMP-9 mRNA expression was determined by real-time PCR. (**E**) Cells were pretreated with GM6001 (1 μM) or MMP2/9 inhibitor (MMP2/9i, 1 μM) for 1 h and then incubated with LPS (2 μg/mL) for 48 h, and cell migration was assayed (magnification = 40×). Data are expressed as mean ± SEM of three independent experiments. * *p* < 0.05; ^#^ *p* < 0.01, as compared with the cells exposed to vehicle or LPS as indicated.

First, we evaluated whether LPS could induce MMP-9 expression. As shown in Figure 1A, LPS induced MMP-9 expression in a time- and concentration-dependent manner. A maximal expression of MMP-9 was found with 2 μg/mL LPS treatment for 24 h in RBA-1 cells. In addition, we used a real-time PCR to determine the level of MMP-9 mRNA expression induced by LPS (2 μg/mL) in RBA-1 cells. MMP-9 mRNA was induced by LPS in a time-dependent manner and reaching a maximal response within 12 h (Figure 1B, open bars). LPS-induced MMP-9 expression was confirmed by a promoter activity assay (Figure 1B, filled bars). To further determine if LPS-induced MMP-9 expression required transcription or translation process, cells were incubated with LPS (2 μg/mL) in the absence or presence of a transcriptional level inhibitor, actinomycin D (Act. D) or a translational level inhibitor, cycloheximide (CHI). As shown in Figure 1C, LPS-induced MMP-9 protein expression was concentration-dependently attenuated by either Act. D or CHI. Moreover, LPS-induced MMP-9 mRNA expression was also inhibited by Act. D, but not by CHI (Figure 1D). These findings demonstrated that the induction of MMP-9 by LPS depends on de novo protein synthesis in RBA-1 cells. MMP-9 has been shown to promote cell migration [22,23]. Thus, to determine whether LPS could induce cell migration via MMP-9 induction, RBA-1 cells were challenged by LPS for 48 h. As shown in Figure 1E, LPS indeed triggered the RBA-1 cell migration, which was blocked by MMP-9 inhibitors, including GM6001 and MMP-9/2 inhibitor. These results indicated that LPS induced cell migration through MMP-9 induction in RBA-1 cells.

### 2.2. LPS Induced proMMP-9 Expression via TLR4

**Figure 2:**
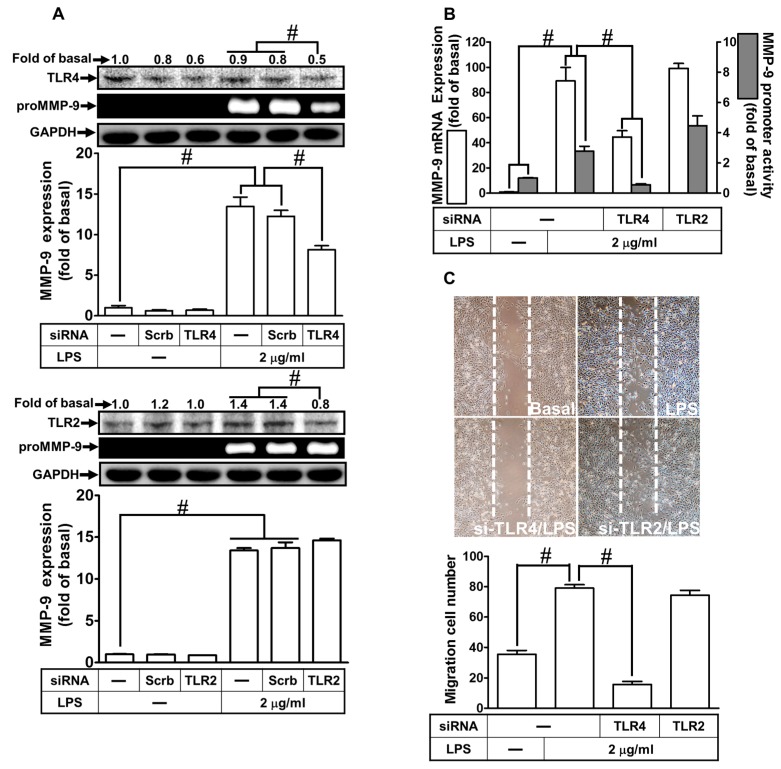
LPS induced the MMP-9 expression and cell migration via Toll-like receptor 4 (TLR4). (**A**) RBA-1 cells were transfected with scrambled (Scrb), TLR4, or TLR2 siRNA, and then incubated with LPS (2 μg/mL) for 24 h. The levels of MMP-9 were determined by gelatin zymography. The GAPDH level of cell lysates was assayed by western blot. (**B**) Cells were transfected with TLR4 or TLR2 siRNA, and then incubated with LPS (2 μg/mL) of 4 h for mRNA expression or 6 h for promoter activity. The mRNA expression and promoter activity of MMP-9 were determined by real-time PCR and promoter assay, respectively. (**C**) Cells were transfected with TLR4 or TLR2 siRNA and then incubated with LPS (2 μg/mL) for 48 h. The number of cell migrations was determined (magnification = 40×). Data are expressed as mean ± SEM of three independent experiments. ^#^ *p* < 0.01 as compared with the cells exposed to vehicle or LPS, as indicated.

LPS has been shown to mediate effects through activation of a family of TLRs on various types of cells. Among these receptors, TLR2 and 4 are activated by LPS [24]. Thus, we investigated whether LPS-induced MMP-9 expression is mediated through TLR2 or TLR4 in RBA-1 cells. As shown in Figure 2A, LPS-induced MMP-9 expression was inhibited by transfection with TLR4 siRNA, but not with TLR2. We further clarified whether TLR2 and 4 were involved in LPS-induced MMP-9 mRNA expression and promoter activity. As shown in Figure 2B, transfection with TLR4 siRNA down-regulated TLR4 protein and subsequently blocked the LPS-induced MMP-9 mRNA expression and promoter activity. Transfection with TLR2 siRNA had no significant effect in the LPS-mediated MMP-9 expression. We further investigated whether LPS-induced cell migration was mediated by TLR4. We found that LPS-induced cell migration was mitigated by transfection with TLR4 siRNA, but not by TLR2 siRNA (Figure 2C). These results suggest that LPS induces MMP-9 expression and cell migration via TLR4 in RBA-1 cells.

### 2.3. LPS-Induced proMMP-9 Expression Was Mediated via c-Src Activation

**Figure 3:**
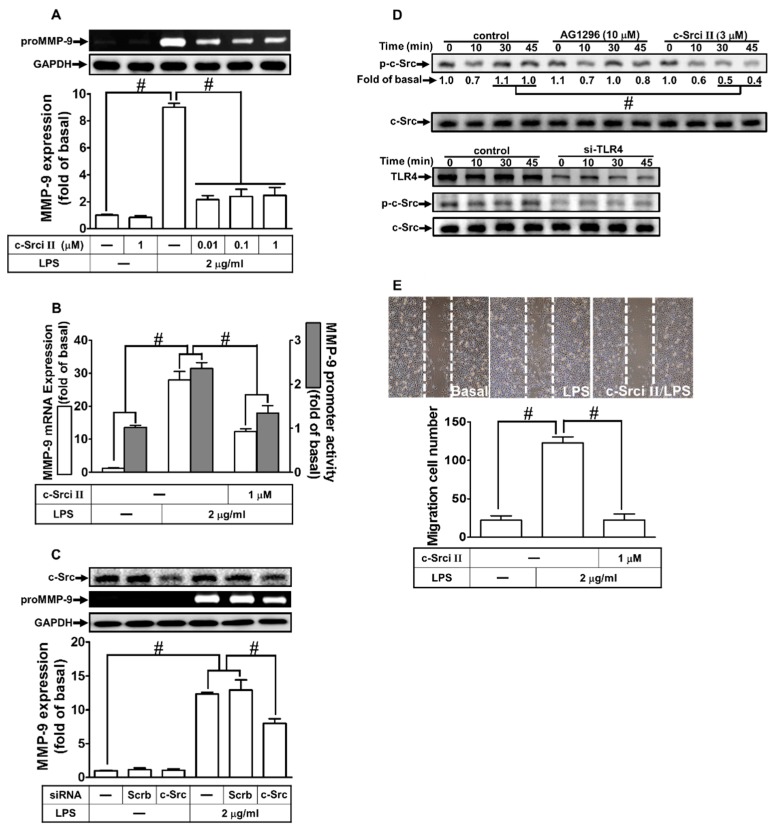
Proto-oncogene tyrosine-protein kinase (c-Src) was involved in LPS-induced MMP-9 expression and cell migration. (**A**) RBA-1 cells were pretreated with c-Src inhibitor II (0.01, 0.1, and 1 μM) for 1 h, and then stimulated with LPS (2 μg/mL) for 24 h. The levels of MMP-9 were examined by gelatin zymography. The GAPDH level of cell lysates was assayed by western blot. (**B**) Cells were pretreated with c-Src inhibitor II (1 μM) for 1 h, and then incubated with LPS (2 μg/mL) of 4 h for mRNA expression or 6 h for promoter activity. The mRNA expression and promoter activity of MMP-9 were determined by real-time PCR and promoter assay, respectively. (**C**) Cells were transfected with scrambled (Scrb) or c-Src siRNA, and then incubated with LPS (2 μg/mL) for 24 h. The medium and cell lysates were collected to respectively determine the levels of MMP-9 by gelatin zymography, and the levels of GAPDH and c-Src by western blotting. (**D**) Cells were pretreated with or without c-Src inhibitor II (3 μM) or AG1296 (10 μM) for 1 h, or separately transfected with TLR4 siRNA, and then challenged with LPS (2 μg/mL) for the indicated time intervals (0, 10, 30, and 45 min). The phosphorylation of c-Src was determined by western blotting. (**E**) Cells were pretreated with c-Src inhibitor II (1 μM) for 1 h, and then incubated with LPS (2 μg/mL) for 48 h. The number of cell migrations was determined (magnification = 40×). Data are expressed as mean ± SEM of three independent experiments. ^#^ *p* < 0.01 as compared with the cells exposed to vehicle or LPS, as indicated.

c-Src is a member of non-receptor tyrosine kinases, which is activated by various stimuli to modulate physiological and pathological functions, such as cell migration and proliferation [25]. We previously demonstrated that c-Src is involved in the LPS-induced ICAM-1 expression [18]. Thus, we evaluated whether LPS-induced MMP-9 expression was mediated through c-Src in RBA-1 cells. Here, our observation demonstrated that LPS-induced MMP-9 protein and mRNA expression, as well as promoter activity, were concentration-dependently attenuated by an inhibitor of c-Src (c-Src inhibitor II) (Figure 3A,B). To further clarify the crucial role of c-Src in the LPS-induced MMP-9 expression, as shown in Figure 3C, transfection with c-Src siRNA down-regulated c-Src protein level and markedly attenuated the LPS-induced MMP-9 expression. Moreover, we investigated whether c-Src phosphorylation was necessary for the LPS-induced MMP-9 expression. As shown in Figure 3D, LPS stimulated c-Src phosphorylation in a time-dependent manner, which was attenuated by pretreatment with c-Src inhibitor II or transfection with TLR4 siRNA, but not by AG1296. Finally, we investigated whether c-Src participated in the LPS-induced cell migration. As shown in Figure 3E, LPS-stimulated cell migration was reduced by pretreatment with c-Src inhibitor II. These results suggest that LPS induces MMP-9 expression and cell migration via a TLR4-dependent c-Src pathway in RBA-1 cells.

### 2.4. LPS Induced proMMP-9 Expression via Pyk2 Phosphorylation

**Figure 4:**
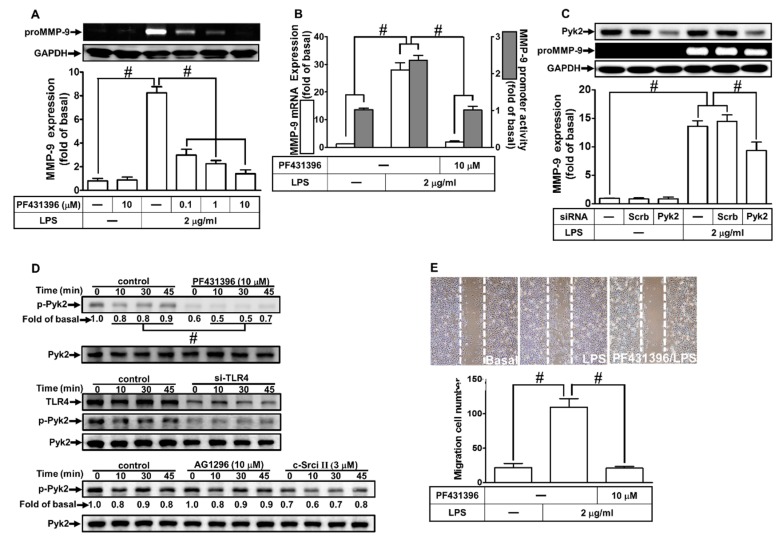
Proline-rich tyrosine kinase 2 (Pyk2) is involved in LPS-induced MMP-9 expression and cell migration. (**A**) RBA-1 cells were pretreated with PF431396 (0.1, 1, and 10 μM) for 1 h, and then incubated with LPS (2 μg/mL) for 24 h. The levels of MMP-9 were determined by gelatin zymography. The GAPDH level of cell lysates was assayed by western blot. (**B**) Cells were pretreated with PF431396 (10 μM) for 1 h, and then incubated with LPS (2 μg/mL) of 4 h for mRNA expression or 6 h for promoter activity. The mRNA expression and promoter activity of MMP-9 were determined by real-time PCR and promoter assay, respectively. (**C**) Cells were transfected with scrambled (Scrb) or Pyk2 siRNA, and then incubated with LPS (2 μg/mL) for 24 h. The medium and cell lysates were collected to respectively determine the levels of MMP-9 by gelatin zymography, and the levels of GAPDH and Pyk2 by western blotting. (**D**) Cells were pretreated with or without PF431396 (10 μM), c-Src inhibitor II (3 μM), or AG1296 (10 μM) for 1 h, or separately transfected with TLR4 siRNA, and then challenged with LPS (2 μg/mL) for the indicated time intervals (0, 10, 30, and 45 min). The phosphorylation of Pyk2 was determined by western blotting. (**E**) Cells were pretreated with PF431316 (10 μM) for 1 h and then incubated with LPS (2 μg/mL) for 48 h. The number of cell migrations was determined (magnification = 40×). Data are expressed as mean ± SEM of three independent experiments. ^#^ *p* < 0.01 as compared with the cells exposed to vehicle or LPS, as indicated.

Our previous study has shown that MMP-9 expression is regulated by Pyk2 phosphorylation in SK-N-SH cells [22]. Thus, we investigated whether Pyk2 was involved in the LPS-induced MMP-9 expression in RBA-1 cells. As shown in Figure 4A,B, pretreatment with PF431396 (an inhibitor of Pyk2) reduced the LPS-induced MMP-9 protein and mRNA expression, as well as the promoter activity. Moreover, transfection with Pyk2 siRNA down-regulated the total protein expression of Pyk2 and attenuated the LPS-induced MMP-9 expression in RBA-1 cells (Figure 4C). We also determined whether LPS-stimulated Pyk2 phosphorylation was required for MMP-9 expression. As shown in Figure 4D, LPS stimulated Pyk2 phosphorylation in a time-dependent manner, which was reduced by pretreatment with PF431396 or c-Src inhibitor II, or separately by transfection with TLR4 siRNA, but not by AG1296. Finally, we investigated whether Pyk2 was involved in the LPS-induced cell migration. As shown in Figure 4E, LPS-stimulated cell migration was reduced by pretreatment with PF431396. These results suggest that LPS induces MMP-9 expression and cell migration via a TLR4-dependent c-Src/Pyk2 pathway in RBA-1 cells.

### 2.5. LPS Induced proMMP-9 Expression through PDGFR Activation

**Figure 5:**
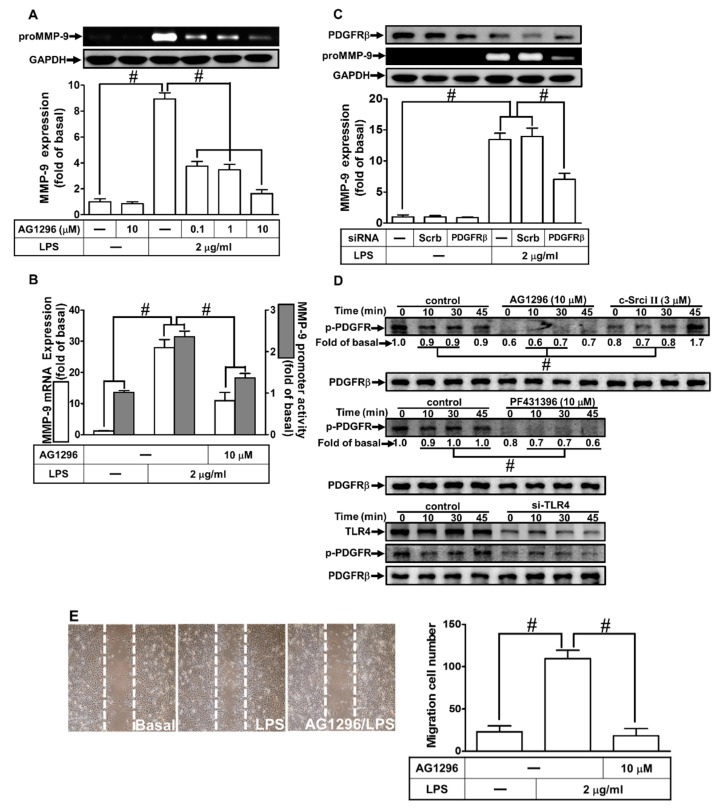
Platelet-derived growth factor receptor (PDGFR)β was involved in LPS-induced MMP-9 expression and cell migration. (**A**) RBA-1 cells were pretreated with AG1296 (0.1, 1, and 10 μM) for 1 h, and then incubated with LPS (2 μg/mL) for 24 h. The levels of MMP-9 were determined by gelatin zymography. The GAPDH level of cell lysates was assayed by western blot. (**B**) Cells were pretreated with AG1296 (10 μM) for 1 h, and then incubated with LPS (2 μg/mL) for 4 h for mRNA expression or 6 h for promoter activity. The mRNA expression and promoter activity of MMP-9 were determined by real-time PCR and promoter assay, respectively. (**C**) Cells were transfected with scrambled (Scrb) or PDGFRβ siRNA, and then incubated with LPS (2 μg/ml) for 24 h. The medium and cell lysates were collected to respectively determine the levels of MMP-9 by gelatin zymography, and the levels of GAPDH and PDGFRβ by western blotting. (**D**) Cells were pretreated with or without AG1296 (10 μM), PF431396 (10 μM), or c-Src inhibitor II (3 μM) for 1 h, or separately transfected with TLR4 siRNA, and then challenged with LPS (2 μg/mL) for the indicated time intervals (0, 10, 30, and 45 min). The phosphorylation of PDGFRβ was determined by western blotting. (**E**) Cells were pretreated with AG1296 (10 μM) for 1 h, and then incubated with LPS (2 μg/mL) for 48 h. The number of cell migrations was determined (magnification = 40×). Data are expressed as mean ± SEM of three independent experiments. ^#^ *p* < 0.01 as compared with the cells exposed to vehicle or LPS, as indicated.

Several studies have shown that PDGFR is a downstream component of c-Src/Pyk2-mediated MMP-9 expression in various types of cells [26,27]. Therefore, we investigated whether PDGFR was involved in the LPS-induced MMP-9 expression in RBA-1 cells. As shown in Figure 5A,B, pretreatment with AG1296 (an inhibitor of PDGFR) markedly reduced the LPS-induced MMP-9 protein in a concentration-dependent manner. In addition, pretreatment with AG1296 also inhibited the LPS-induced MMP-9 mRNA expression and promoter activity (Figure 5B). To further confirm the role of PDGFR in LPS-induced MMP-9 expression, transfection with PDGFR siRNA reduced PDGFR protein level and inhibited the LPS-induced MMP-9 expression in RBA-1 cells (Figure 5C). We also clarified whether LPS-stimulated PDGFR phosphorylation was necessary for MMP-9 expression in RBA-1 cells. As shown in Figure 5D, LPS stimulated PDGFR phosphorylation in a time-dependent manner, which was inhibited by pretreatment with AG1296, PF431396, and c-Src inhibitor II, or transfection with TLR4 siRNA. Finally, pretreatment with AG1296 also attenuated cell migration induced by LPS (Figure 5E). These results suggest that LPS-induced MMP-9 expression is mediated through a TLR4-dependent c-Src/Pyk2/PDGFR pathway in RBA-1 cells.

### 2.6. PI3K/Akt Were Involved in LPS-Induced proMMP-9 Expression

**Figure 6:**
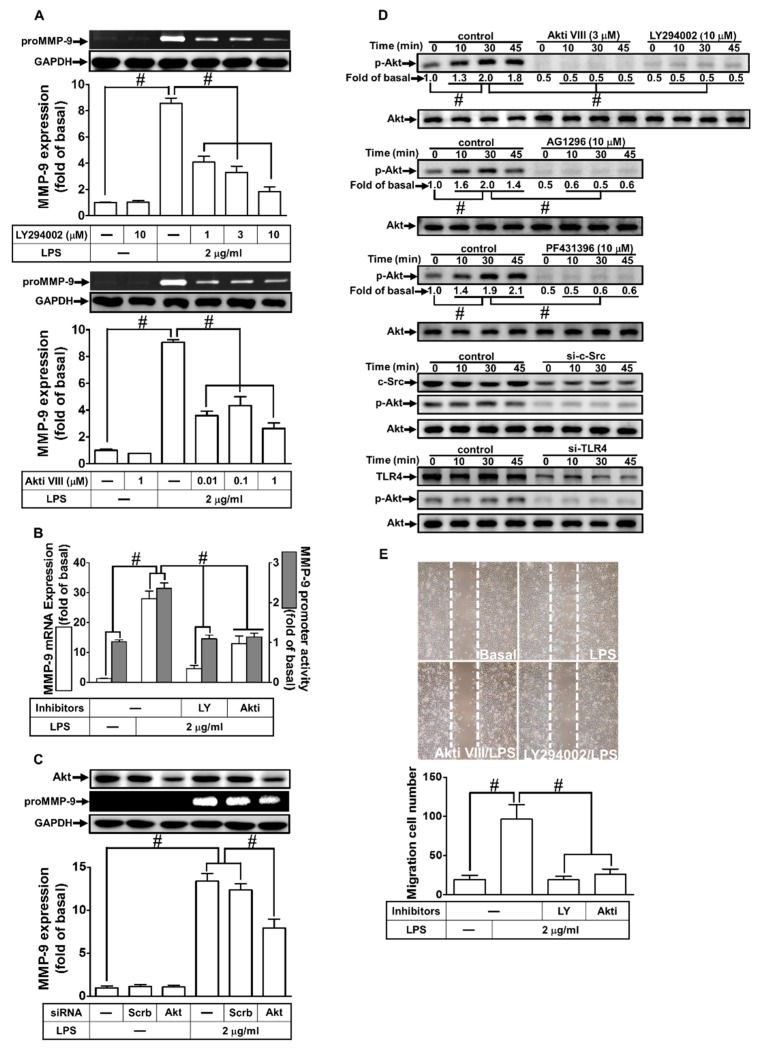
Phosphoinositide 3-kinase (PI3K)/protein kinase B (Akt) pathway was involved in LPS-induced MMP-9 expression and cell migration. (**A**) RBA-1 cells were pretreated with LY294002 (1, 3, and 10 μM) or Akt inhibitor VIII (0.01, 0.1, and 1 μM) for 1 h, independently, and then incubated with LPS (2 μg/mL) for 24 h. The levels of MMP-9 were determined by gelatin zymography. The GAPDH level of cell lysates was assayed by western blot. (**B**) Cells were pretreated with LY294002 (10 μM) or Akt inhibitor VIII (1 μM) for 1 h, independently, and then incubated with LPS (2 μg/mL) for 4 h for mRNA expression or 6 h for promoter activity. The mRNA expression and promoter activity of MMP-9 were determined by real-time PCR and promoter assay, respectively. (**C**) Cells were transfected with scrambled (Scrb) or Akt siRNA, and then incubated with LPS (2 μg/mL) for 24 h. The medium and cell lysates were collected to respectively determine the levels of MMP-9 by gelatin zymography, and the levels of GAPDH and Akt by western blotting. (**D**) Cells were pretreated with or without LY294002 (10 μM), AKT inhibitor VIII (3 μM), AG1296 (10 μM), or PF431396 (10 μM) for 1 h, or separately transfected with either TLR4 or c-Src siRNA, and then challenged with LPS (2 μg/mL) for the indicated time intervals (0, 10, 30, and 45 min). The phosphorylation of Akt was determined by western blotting. (**E**) Cells were pretreated with Akt inhibitor VIII (1 μM) or LY294002 (3 μM) for 1 h, and then incubated with LPS (2 μg/mL) for 48 h. The number of cell migrations was determined (magnification = 40×). Data are expressed as mean ± SEM of three independent experiments. ^#^ *p* < 0.01 as compared with the cells exposed to vehicle or LPS, as indicated.

Akt is a serine/threonine protein kinase that modulates several cellular processes, such as metabolism, survival, and proliferation in various types of cells [28]. Moreover, PI3K/Akt have been shown to mediate MMP-9 expression induced by various stimuli [11,22]. To investigate whether PI3K/Akt were involved in the LPS-induced MMP-9 expression in RBA-1 cells, we used the inhibitor of PI3K (LY294002) or Akt (Akt inhibitor VIII) for these purposes. We found that pretreatment with either LY294002 or Akt inhibitor VIII reduced LPS-induced MMP-9 protein and mRNA expression, as well as promoter activity (Figure 6A,B). To further confirm the role of Akt in LPS-mediated MMP-9 expression, as shown in Figure 6C, transfection with Akt siRNA down-regulated Akt protein level and then reduced the LPS-induced MMP-9 expression in RBA-1 cells. We also investigated whether LPS-stimulated Akt phosphorylation was necessary for MMP-9 expression. As shown in Figure 6D, LPS stimulated Akt phosphorylation in a time-dependent manner, which was inhibited by pretreatment with Akt inhibitor VIII or LY294002. We also differentiated the relationship among Akt, c-Src, Pyk2, and PDGFR in the LPS-mediated responses. As shown in Figure 6E, LPS-stimulated Akt phosphorylation was inhibited by pretreatment with AG1296, PF431396, and c-Src inhibitor II, or transfection with TLR4 siRNA in RBA-1 cells. In addition, pretreatment with either LY294002 or Akt inhibitor VIII also attenuated the LPS-enhanced cell migration (Figure 6E). These results suggest that LPS-induced MMP-9 expression is mediated through a TLR4/c-Src/Pyk2/PDGFR/PI3K/Akt pathway in RBA-1 cells.

### 2.7. LPS Induced proMMP-9 Expression via MAPKs

**Figure 7:**
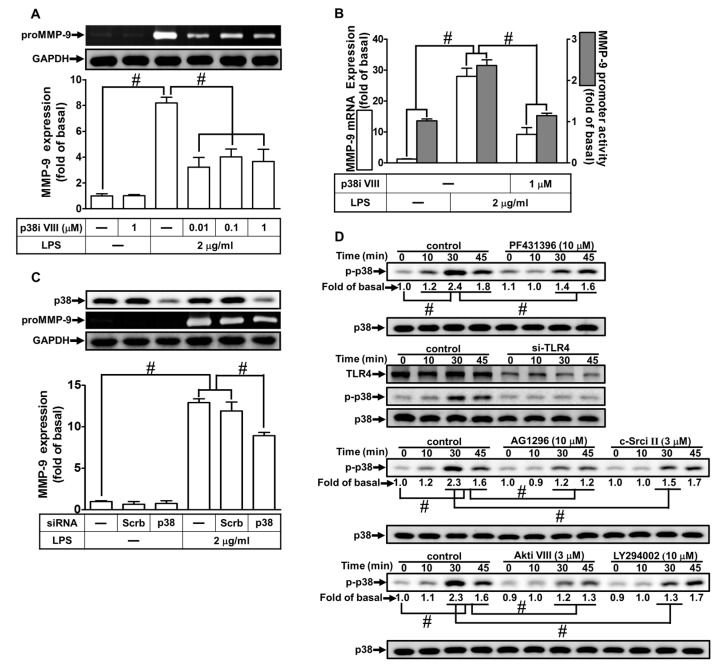

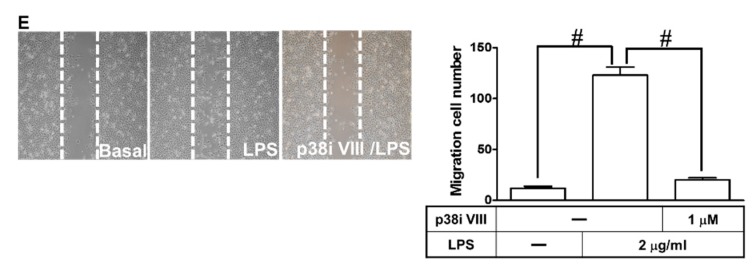
p38 mitogen-activated protein kinase (MAPK) was involved in LPS-induced MMP-9 expression and cell migration. (**A**) RBA-1 cells were pretreated with p38 MAP kinase inhibitor VIII (0.01, 0.1, and 1 μM) for 1 h, and then incubated with LPS (2 μg/mL) for 24 h. The levels of MMP-9 were determined by gelatin zymography. The GAPDH level of cell lysates was assayed by western blot. (**B**) Cells were pretreated with p38 MAP kinase inhibitor VIII (1 μM) for 1 h, and then incubated with LPS (2 μg/mL) for 4 h for mRNA expression or 6 h for promoter activity. The mRNA expression and promoter activity of MMP-9 were determined by real-time PCR and promoter assay, respectively. (**C**) Cells were transfected with scrambled (Scrb) or p38 siRNA, and then incubated with LPS (2 μg/mL) for 24 h. The medium and cell lysates were collected to respectively determine the levels of MMP-9 by gelatin zymography, and the levels of GAPDH and p38 MAPK by western blotting. (**D**) Cells were pretreated with or without p38 MAP kinase inhibitor VIII (3 μM), AG1296 (10 μM), PF431396 (10 μM), Akt inhibitor VIII (3 μM), LY294002 (10 μM), or c-Src inhibitor II (3 μM) for 1 h, or separately transfected with TLR4 siRNA, and then challenged with LPS (2 μg/mL) for the indicated time intervals (0, 10, 30, and 45 min). The phosphorylation of p38 MAPK was determined by western blotting. (**E**) Cells were pretreated with p38 MAP kinase inhibitor VIII (1 μM) for 1 h, and then incubated with LPS (2 μg/mL) for 48 h. The number of cell migrations was determined (magnification = 40×). Data are expressed as mean ± SEM of three independent experiments. ^#^ *p* < 0.01 as compared with the cells exposed to vehicle or LPS, as indicated.

**Figure 8:**
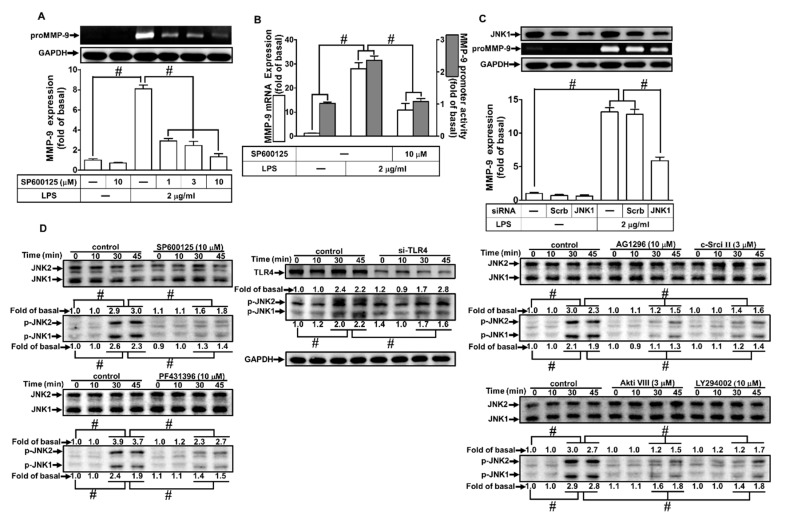

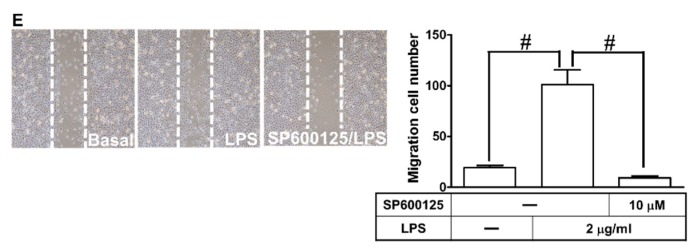
Jun amino-terminal kinase (JNK)1/2 were involved in LPS-induced MMP-9 expression and cell migration. (**A**) RBA-1 cells were pretreated with SP600125 (1, 3, and 10 μM) for 1 h, and then incubated with LPS (2 μg/mL) for 24 h. The levels of MMP-9 were determined by gelatin zymography. The GAPDH level of cell lysates was assayed by western blot. (**B**) Cells were pretreated with SP600125 (10 μM) for 1 h, and then incubated with LPS (2 μg/mL) for 4 h for mRNA expression or 6 h for promoter activity. The mRNA expression and promoter activity of MMP-9 were determined by real-time PCR and promoter assay, respectively. (**C**) Cells were transfected with scrambled (Scrb) or JNK1 siRNA, and then incubated with LPS (2 μg/mL) for 24 h. The medium and cell lysates were collected to respectively determine the levels of MMP-9 by gelatin zymography, and the levels of GAPDH and JNK1 by western blotting. (**D**) Cells were pretreated with or without SP600125 (10 μM), c-Src inhibitor II (3 μM), AG1296 (10 μM), PF431396 (10 μM), Akt inhibitor VIII (3 μM), or LY294002 (10 μM) for 1 h, or separately transfected with TLR4 siRNA, and then challenged with LPS (2 μg/mL) for the indicated time intervals (0, 10, 30, and 45 min). The phosphorylation of JNK1/2 was determined by western blotting. (**E**) RBA-1 cells were pretreated with SP600125 (3 μM) for 1 h, and then incubated with LPS (2 μg/mL) for 48 h. The number of cell migrations was determined (magnification = 40×). Data are expressed as mean ± SEM of three independent experiments. ^#^*p* < 0.01 as compared with the cells exposed to vehicle or LPS, as indicated.

MAPKs play crucial roles in cellular functions, such as cell proliferation, differentiation, migration, senescence, and apoptosis [29]. Several reports have demonstrated that activation of MAPKs causes the expression of various inflammatory genes, including MMP-9 [11,30]. Therefore, we explored whether MAPKs contributed to the LPS-induced MMP-9 expression. As shown in Figure 7A,B, and Figure 8A,B, pretreatment with the inhibitor of either p38 MAPK (p38 MAP kinase inhibitor VIII) or JNK1/2 (SP600125) markedly attenuated LPS-induced MMP-9 protein, mRNA levels, and promoter activity. To confirm these results, we found that transfection with JNK1 or p38 MAPK siRNA reduced the expression of JNK1 or p38 MAPK, respectively, and then attenuated the LPS-induced MMP-9 expression (Figure 7C and Figure 8C). Moreover, LPS also markedly stimulated p38 MAPK and JNK1/2 phosphorylation in a time-dependent manner, which was inhibited by pretreatment with p38 MAP kinase inhibitor VIII or SP600125, respectively (Figure 7D and Figure 8D). Moreover, we also differentiated the relationship among MAPKs, Akt, c-Src, Pyk2, PDGFR, and TLR4 in LPS-mediated responses. As shown in Figure 7D and Figure 8D, LPS-stimulated p38 MAPK and JNK1/2 phosphorylation were inhibited by pretreatment with Akt inhibitor VIII, AG1296, PF431396, or c-Src inhibitor II, or transfection with TLR4 siRNA in RBA-1 cells. Finally, as shown in Figure 7E and Figure 8E, pretreatment with either p38 MAP kinase inhibitor VIII or SP600125 markedly attenuated the LPS-enhanced cell migration in RBA-1 cells. These results suggest that LPS-induced MMP-9 expression is mediated through a TLR4/c-Src/Pyk2/PDGFR/PI3K/Akt-dependent p38 MAPK and JNK1/2 pathways in RBA-1 cells.

### 2.8. LPS Induced proMMP-9 Expression via AP-1

**Figure 9:**
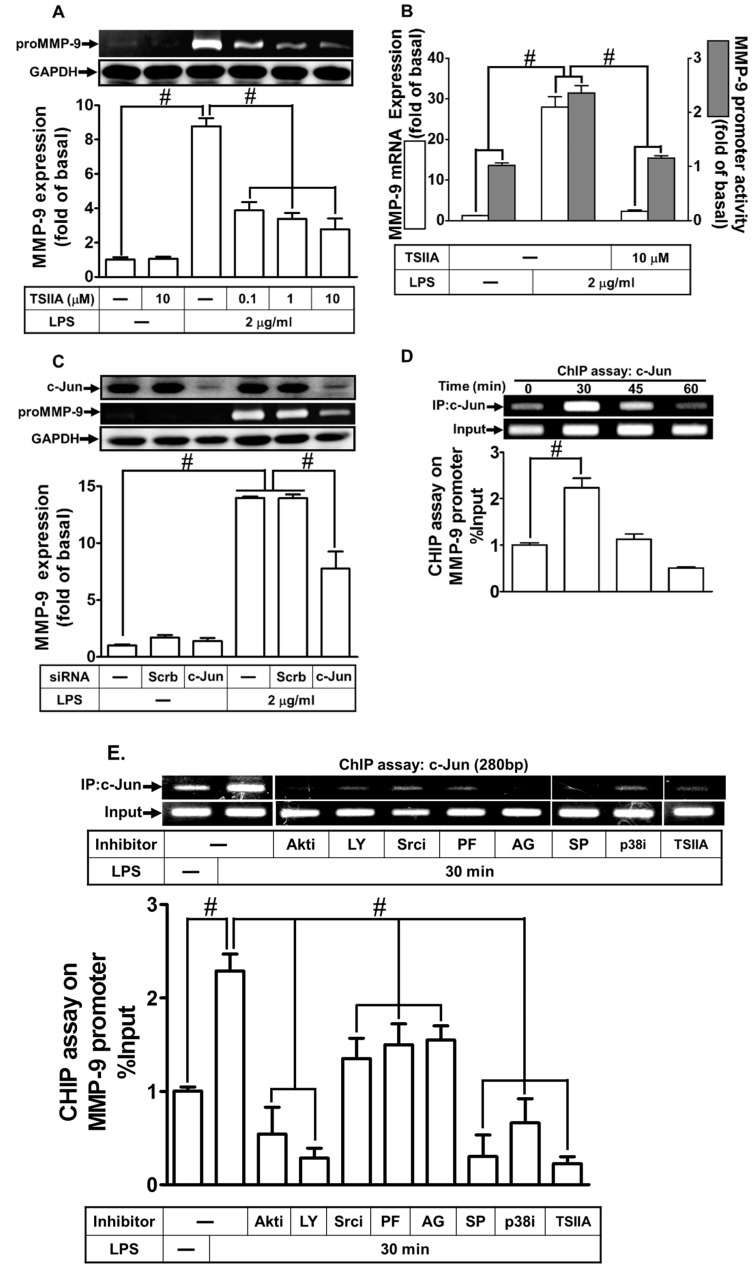

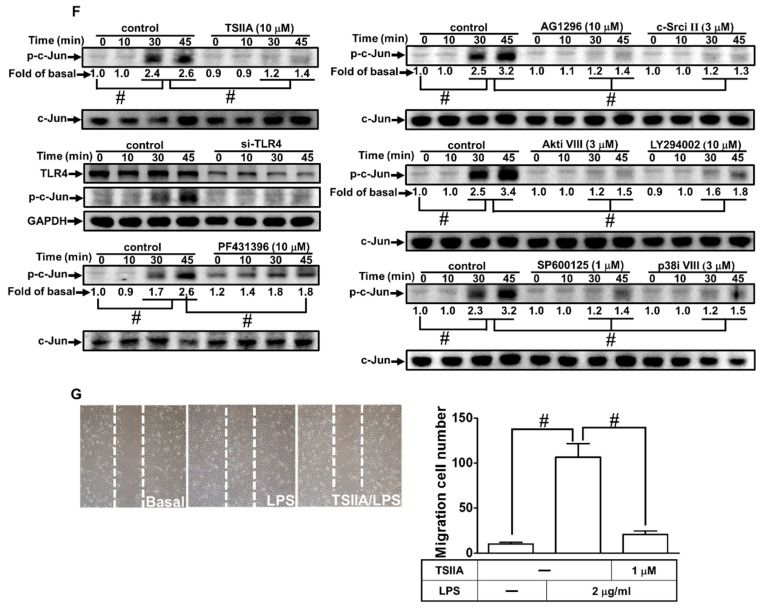
Activator protein 1 (AP-1) was necessary for LPS-induced MMP-9 expression and cell migration. (**A**) RBA-1 cells were pretreated with tanshinone IIA (0.1, 1, and 10 μM) for 1 h, and then incubated with LPS (2 μg/mL) for 24 h. The levels of MMP-9 were determined by gelatin zymography. The GAPDH level of cell lysates was assayed by western blot. (**B**) Cells were pretreated with tanshinone IIA (10 μM) for 1 h, and then incubated with LPS (2 μg/mL) for 4 h for mRNA expression or 6 h for promoter activity. The mRNA expression and promoter activity of MMP-9 were determined by real-time PCR and promoter assay, respectively. (**C**) Cells were transfected with scrambled (Scrb) or c-Jun (a member of the transcription factor AP-1 family) siRNA, and then incubated with LPS (2 μg/mL) for 24 h. The medium and cell lysates were collected to respectively determine the levels of MMP-9 by gelatin zymography, and the levels of GAPDH and c-Jun by western blotting. (**D**) Cells were treated with LPS (2 μg/mL) for the indicated time intervals (0, 30, 45, and 60 min). (**E**) Cells were pretreated with Akt inhibitor VIII (Akti, 3 μM), LY294002 (LY, 10 μM), c-Src inhibitor II (Srci, 3 μM), PF431396 (PF, 10 μM), AG1296 (AG, 10 μM), tanshinone IIA (TSIIA, 10 μM), SP600125 (SP, 1 μM), or p38 MAP kinase inhibitor VIII (p38i, 3 μM) for 1 h, and then challenged with LPS (2 μg/mL) for 30 min. (**D**,**E**) The levels of c-Jun binding to MMP-9 promoter were determined by a chromatin immunoprecipitation (ChIP) assay. To fit the construct of data layout, the representational images of ChIP assay were rearranged from the same piece of gel, except for non-related inhibitors, and disclosed by the insertion of white spaces. (**F**) Cells were pretreated with or without tanshinone IIA (10 μM), PF431396 (10 μM), AG1296 (10 μM), c-Src inhibitor II (3 μM), Akt inhibitor VIII (3 μM), LY294002 (10 μM), SP600125 (1 μM), or p38 MAP kinase inhibitor VIII (3 μM) for 1 h, or separately transfected with TLR4 siRNA, and then challenged with LPS (2 μg/mL) for the indicated time intervals (0, 10, 30, and 45 min). The phosphorylation of c-Jun was determined by western blotting. (**G**) Cells were pretreated with tanshinone IIA (1 μM) for 1 h, and then incubated with LPS (2 μg/mL) for 48 h. The number of cell migrations was determined (magnification = 40×). Data are expressed as mean ± SEM of three independent experiments. ^#^ *p* < 0.01 as compared with the cells exposed to vehicle or LPS, as indicated.

Several reports have indicated that activation of AP-1 leads to the expression of various inflammatory genes, including MMP-9 [11,22,31]. In addition, the promoter region of MMP-9 consists of AP-1 binding sites that are regulated by various stimuli in different types of cells [11,22]. Therefore, we investigated whether LPS-induced MMP-9 expression was mediated through AP-1 in RBA-1 cells. As shown in Figure 9A,B, retreatment with tanshinone IIA (1,6,6-Trimethyl-6,7,8,9-tetrahydrophenanthro[1,2-b]furan-10,11-dione, an AP-1 inhibitor) markedly reduced the LPS-induced proMMP-9 protein and mRNA expression, as well as the promoter activity. Among AP-1 subfamily, c-Jun (a member of the transcription factor AP-1 family) is an important transcriptional activator that combines with c-Fos (a member of the transcription factor AP-1 family) to directly activate genes including MMP transcription by binding on its promoter AP-1 motifs [11]. To further confirm the role of AP-1 in the LPS-induced MMP-9 expression, as shown in Figure 9C, transfection with c-Jun siRNA was found to downregulate the protein expression of c-Jun, inhibiting the LPS-induced proMMP-9 expression. To further ensure the role of AP-1 in the LPS-mediated MMP-9 induction, we applied chromatin immunoprecipitation (ChIP) assay to clarify whether LPS-stimulated recruitment of c-Jun to MMP-9 promoter was involved in MMP-9 gene expression. As shown in Figure 9D, LPS stimulated the binding of c-Jun to the MMP-9 promoter in a time-dependent manner. This stimulatory effect was attenuated by pretreatment with Akt inhibitor VIII, LY294002, c-Src inhibitor II, PF431396, AG1296, tanshinone IIA, SP600125, or p38 MAP kinase inhibitor VIII (Figure 9E). Moreover, we also investigated whether LPS-stimulated c-Jun phosphorylation was necessary for MMP-9 expression. As shown in Figure 9E, LPS markedly stimulated c-Jun phosphorylation in a time-dependent manner, which was inhibited by pretreatment with tanshinone IIA. Moreover, we also investigated whether c-Jun phosphorylation was mediated through a TLR4/c-Src/Pyk2/PDGFR/PI3K/Akt/p38 MAPK and JNK1/2-dependent pathway. As shown in Figure 9F, LPS-stimulated c-Jun phosphorylation was inhibited by pretreatment with p38 MAP kinase inhibitor VIII, SP600125, AKT inhibitor VIII, AG1296, PF431396, and c-Src inhibitor II, or transfection with TLR4 siRNA in RBA-1 cells. Finally, pretreatment with tanshinone IIA markedly reduced the LPS-induced cell migration (Figure 9G). These results suggest that LPS-induced MMP-9 expression is mediated through a TLR4/c-Src/Pyk2/PDGFR/PI3K/Akt/p38 MAPK and JNK1/2-dependent AP-1 activation pathway in RBA-1 cells.

## 3. Discussion

**Figure 10:**
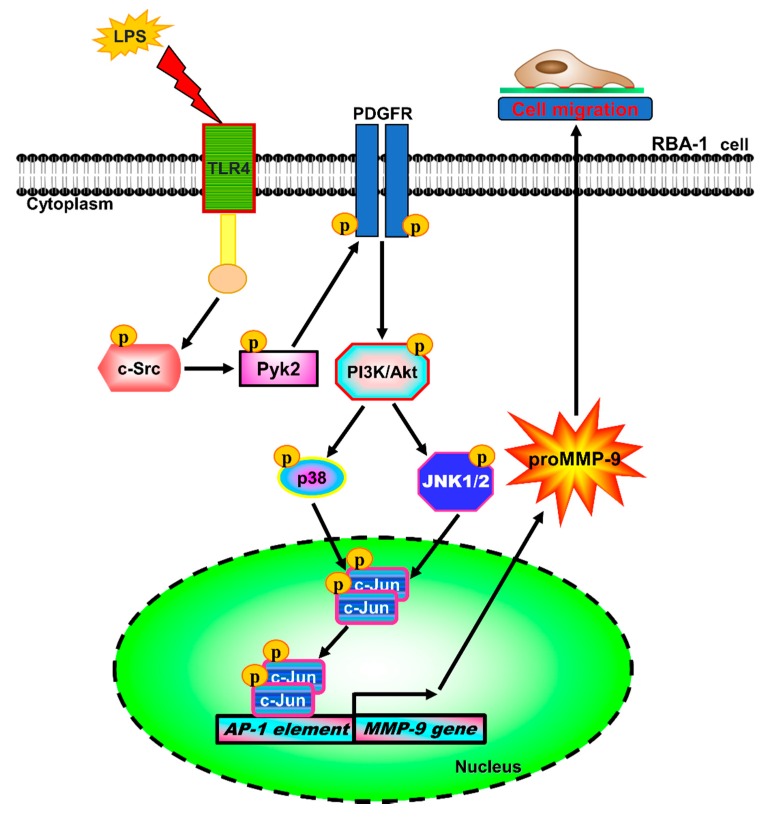
Schematic signaling pathways were involved in MMP-9 expression and cell migration in RBA-1 cells challenged with LPS. Our results demonstrated that LPS-induced MMP-9 expression and cell migration were mediated through a TLR4/c-Src/Pyk2/PDGFRβ/PI3k/AKT/p38 MAPK and JNK1/2-dependent cascade to activate AP-1 in RBA-1 cells. 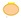: Toll/IL-1 receptor (TIR) domain.

MMPs, especially MMP-9, participate in the pathological processes of demyelination, neuroinflammation, and neurotoxicity in the brain, associated with many CNS diseases [8,9,10]. LPS is a pathogen of neuroinflammatory diseases in human and animal models [32,33]. The pathological processes are, at least partially, mediated through LPS, which activates astrocytes to release pro-inflammatory proteins, including MMP-9, or induces astrocyte proliferation [34,35]. Moreover, neuroinflammation is a key factor in neurodegeneration [36]. These findings suggest that LPS, MMP-9, and reactive astrocytes may be closely involved in the development of neurodegenerative processes. However, the molecular mechanisms underlying LPS-induced MMP-9 expression are not fully understood in cultured RBA-1 cells. The present study demonstrated that MMP-9 expression associated with cell migration induced by LPS was, at least in part, mediated through a TLR4/c-Src/Pyk2/PDGFR/PI3K/Akt/p38 MAPK and JNK1/2-dependent pathway, leading to activation of AP-1 in RBA-1 cells (Figure 10). These findings suggest that LPS induces brain inflammation via induction of MMP-9 expression, leading to an increase of BBB permeability, recruitment of immune cells, cell migration, and tissue remodeling during brain insults [8].

The effects of LPS on cells are mediated by a family of TLRs on various types of cells [18,20]. Among these TLRs, TLR4 is activated by LPS [24]. Moreover, TLRs expressed by numerous types of cells are involved in different cellular processes, such as growth, development, mitogenesis, atherogenesis, and inflammation. Indeed, we also found that TLR2 and TLR4 were expressed on RBA-1 cells. A previous study has shown that TLR-mediated signalings play a critical role in neurodegenerative diseases, such as Alzheimer′s disease, Parkinson′s disease, amyotrophic lateral sclerosis, and multiple system atrophy [37]. Here, we proved that LPS potentially enhanced MMP-9 expression and cell migration, mainly via TLR4 activation, as transfection with TLR4 but not TLR2 siRNA attenuated the LPS-mediated responses and phosphorylation of downstream signaling molecules. These results are in line with the evidence indicating that TLR4 is activated by both infectious and non-infectious factors in various types of cells [16,17]. The present results are also consistent with the findings that LPS-induced ICAM-1 expression is mediated through a TLR4-dependent pathway [18].

Src family kinases (SFKs) play a key role in the transduction of signals by TLR for the expression of inflammatory genes, such as MMP-9, in various types of cells [21]. Thus, Src plays a crucial role in several cellular functions, including cell migration and proliferation [25]. LPS has been shown to stimulate the phosphorylation of SFKs [18]. These findings are consistent with our present observations, indicating that LPS-induced MMP-9 expression and cell migration are mediated through TLR4-dependent c-Src phosphorylation, as pretreatment with the inhibitors of c-Src (c-Src inhibitor II) attenuated the LPS-mediated responses in RBA-1 cells. Moreover, several signaling components have been demonstrated to be tyrosine phosphorylated by c-Src, such as Pyk2 and PDGFR [18,22]. Our results indicated that Pyk2/PDGFR were downstream signaling components of c-Src, as pretreatment with c-Src inhibitor II attenuated the LPS-stimulated phosphorylation of Pyk2 and PDGFR. These findings are also consistent with the Japanese encephalitis virus and thrombin-mediated responses through c-Src-dependent phosphorylation of Pyk2 and PDGFR in RBA-1 cells and SK-N-SH cells [11,22].

In addition to c-Src, Pyk2 has also been shown to be activated by various stimuli, including LPS, leading to the expression of inflammatory genes [19]. A previous study demonstrated that tyrosine phosphorylation of Pyk2 leads to the binding of SH2 domain of Src to Tyr^402^ of Pyk2 and activation of Src [38]. On the contrary, our previous results demonstrated the different relationship between c-Src and Pyk2 [22,39]. Moreover, Pyk2 has been shown to be involved in the expression of MMP-9 [22]. In the present study, we found that pretreatment with c-Src inhibitor II attenuated phosphorylation of c-Src and Pyk2 stimulated by LPS. These results are consistent with our previous studies, indicating that thrombin-induced MMP-9 and cyclooxygenase (COX)-2 expression are mediated through c-Src/Pyk2 signal cascade in SK-N-SH neuroblastoma and human cardiomyocytes, respectively [26,39]. Thus, we confirmed that LPS-induced MMP-9 expression is mediated through a TLR4/c-Src/Pyk2 pathway in RBA-1 cells.

PDGF/PDGFR are essential for an array of physiological processes, such as cell migration and proliferation. However, deregulated PDGFR activity also contributes to various pathological processes, such as cancer, fibrosis, neurological diseases, and atherosclerosis [40]. Pyk2 has been recognized as an important upstream mediator of PDGFR transactivation involved in the regulation of cell proliferation [41]. In addition, it has been demonstrated that MMP-9 expression is mediated through transactivation of PDGFR induced by IL-1β, TNF-α, and lipoteichoic acid (LTA) [42,43,44]. Furthermore, in an animal model of ischemic stroke, tissue plasminogen activator was found to impair BBB integrity mediated through activation of PDGFR [45]. Here, we confirmed that LPS-induced MMP-9 expression and PDGFR phosphorylation was significantly attenuated by the inhibitors of PDGFR (AG1296), c-Src (c-Src inhibitor II), Pyk2 (PF431396), and transfection with TLR4 siRNA in RBA-1 cells. These findings suggest that LPS-induced MMP-9 expression is mediated through a TLR4/c-Src/Pyk2-dependent PDGFR pathway in RBA-1 cells.

PI3K is a heterodimeric protein consisting of a p85 regulatory subunit and a p110 catalytic subunit. PI3K displays important functions in several cellular processes [28] and the pathogenesis of tumor progression and inflammatory responses [46]. Akt, a downstream component of the PI3K pathway, is also important in the regulation of fundamental cellular functions, including transcription, translation, proliferation, growth, and survival [47]. The activated PDGFR recruits SH-2 (Src Homology 2) domain-containing signal transduction proteins and activates signaling components, including c-Src, PI3K, and phospholipase C (PLC)γ [48]. Several lines of evidence have supported that PI3K/Akt are downstream components of PDGFR activated by different stimuli in various types of cells [18,42]. Moreover, PI3K/Akt are well known as critical regulators of AP-1 and nuclear factor-κB (NF-κB) activation [18,22]. The expression of MMP-9 was previously shown to be modulated by the activation of PI3K and Akt in neuronal cells and astroglia [11,22]. In the present study, we confirmed the roles of PI3K/Akt in LPS-induced MMP-9 expression in RBA-1 cells. Our observations demonstrated that transfection with PDGFR or Akt siRNA inhibited the LPS-induced MMP-9 expression, and LPS-stimulated Akt phosphorylation was inhibited by pretreatment with LY294002 or AG1296. These results confirmed that LPS-induced MMP-9 expression was mediated through PDGFR/PI3K/Akt cascade. Our findings are consistent with the previous results demonstrating that MMP-9 expression is also mediated through a PI3K/Akt cascade in Japanese encephalitis virus- and thrombin-challenged rat brain astrocytes [11].

MAPKs, a group of serine-threonine kinases, are important signaling components that transduce the signaling from the cell surface to the nucleus. MAPKs have been implicated in the regulation of many cellular responses, such as inflammation, proliferation, differentiation, and apoptosis [29]. MAPKs consist of extracellular signal-regulated kinases 1 and 2 (Erk1/2), c-Jun N-terminal kinases 1 to 3 (JNK1 to JNK3), p38 MAPK (α, β, γ, and δ), and Erk5 families [49]. Previous studies have shown that LPS stimulates MAPK activation in various types of cells [50,51]. Moreover, our previous studies and others have indicated that Erk1/2, JNK1/2, and p38 MAPK could regulate MMP-9 induction in various types of cells [11,22,30]. In addition, MAPKs have been shown to be downstream components of PI3K/Akt in various cell types [11,22]. Therefore, we investigated the role of MAPKs in LPS-induced MMP-9 expression in RBA-1 cells. Our results suggested that p38 MAPK and JNK1/2 play a role in the LPS-induced MMP-9 expression in brain astrocytes. Indeed, in RBA-1 cells, downregulation of Akt activity could attenuate LPS-induced phosphorylation of p38 MAPK and JNK1/2, but not Erk1/2. The results are compatible with previous studies indicating that MMP-9 expression was mediated through activation of p38 MAPK and JNK1/2 in various types of cells (11, 26, 34).

The promoter of MMP-9 possesses a series of functional activator element-binding sites, including nuclear factor-κB (NF-κB) and AP-1 [8]. AP-1 activation is stimulated by various factors, including bacterial and viral infections, cytokines, physical and chemical stresses, and thrombin [11,31]. However, AP-1 involved in the LPS-induced MMP-9 expression was poorly understood in RBA-1 cells. We have previously demonstrated that AP-1 participated in Japanese encephalitis virus-induced MMP-9 expression in RBA-1 cells [11]. Our results of this study also confirmed the role of AP-1 in MMP-9 expression by pretreatment with tanshinone IIA in RBA-1 cells challenged with LPS. These results suggest that LPS-induced MMP-9 expression is mediated through AP-1 activation in RBA-1 cells. Further, our data demonstrated that LPS stimulated AP-1 activation via Akt-dependent p38 MAPK and JNK1/2, but not p42/p44 MAPK phosphorylation in RBA-1 cells by pretreatment with Akt inhibitor VIII, LY294002, SP600125, and p38 MAP kinase inhibitor VIII. These results were confirmed by using a ChIP assay. LPS stimulated the recruitment of AP-1 binding to MMP-9 promoter, also attenuated by these pharmacological inhibitors. Interestingly, in human monocytes, LPS stimulation activates a variety of transcription factors, including NF-κB (p50/p65) and AP-1 (c-Fos/c-Jun), through Erk1/2, JNK1/2, and p38 MAPK [52]. In fact, we have found that inhibition of NF-κB activity by Bay11-7082 or transfection with p65 siRNA significantly attenuated the LPS-induced MMP-9 expression, mRNA levels, and promoter activity (data not shown). Thus, we proposed that LPS possibly enhances MMP-9 expression via a MAPK-independent NF-κB pathway. These findings suggest that LPS-induced MMP-9 expression is mediated through a TLR4/c-Src/Pyk2/PDGFR/p38 MAPK and JNK1/2-dependent AP-1 pathway in RBA-1 cells.

In summary, on the basis of our data and the previous literature, we demonstrated a model of the signaling mechanisms implicated in the LPS-mediated MMP-9 expression and cell migration in RBA-1 cells (Figure 10). These results suggest that LPS-induced MMP-9 expression associated with cell migration is mediated through a TLR4/c-Src/Pyk2/ PDGFR/PI3K/Akt/MAPK-dependent AP-1 activation. These findings imply that LPS might play a crucial role in the development of neuroinflammation and neurodegeneration, providing new insights into the mechanisms involved in CNS neuroinflammation triggered by LPS in brain astrocytes. Therefore, our experiments could provide useful targets to develop an effective therapeutic strategy for the management of brain inflammation.

## 4. Materials and Methods

### 4.1. Antibodies and Inhibitors

Dulbecco′s modified Eagle′s medium (DMEM)/Ham′s nutrient mixture F-12 (F-12), fetal bovine serum (FBS), and siRNAs for c-Src (Csk-RSS321555), Akt (RSS301983), c-Jun (RSS240570), PDGFR (RSS351966), P38 (RSS340227), and JNK1 (RSS331962) were purchased from Invitrogen (Carlsbad, CA, USA). Hybond C membrane and enhanced chemiluminescence (ECL) detection system were from GE Healthcare Biosciences (Buckinghamshire, UK). All primary antibodies were diluted at 1:1000 in phosphate-buffered saline (PBS) with 1% bovine serum albumin (BSA). Anti-phospho-Akt (Ser^473^, #9271), anti-phospho-Pyk2 (Tyr^402^, #3291), anti-phospho-PDGFRβ (Tyr^751^, #3161), anti-phospho-p38 MAPK (Thr^180^/Tyr^182^, #9211), anti-phospho-JNK1/2 (Thr^183^/Tyr^185^, #4668), anti-phospho-extracellular signal-regulated kinase (Erk)1/2 (Thr^202^/Tyr^204^, #9101), and anti-phospho-c-Jun (Ser^63^, #2361) antibodies were from Cell Signaling (Danvers, MA, USA). Anti-Pyk2 (ab32448) antibody was from Abcam (Cambridge, United Kingdom). Anti-glyceraldehyde-3-phosphate dehydrogenase (GAPDH) (#MCA-ID4) was from Encor (Gainesville, FL). Anti-TLR2 (sc-10739), anti-TLR4 (sc-16240), anti-PDGFRβ (sc-374573), anti-c-Src (sc-8056), anti-phospho-c-Src (Tyr^139^, sc-12928-R), anti-p38 MAPK (sc-535), anti-Akt (sc-8312), anti-Erk1 (sc-271270), anti-Erk2 (sc-1647), anti-JNK1/2 (sc-7345), and anti-c-Jun (sc-44) antibodies were from Santa Cruz (Santa Cruz, CA, USA). Bicinchoninic acid (BCA) protein assay reagent was from Pierce (Rockford, IL, USA). Sodium dodecyl sulfate-polyacrylamide gel electrophoresis (SDS-PAGE) reagents, and siRNA duplex pool targeted against rat TLR4 (forward 5′-GCAUAGAGGUACUUCCUAA-3′, reverse 5′-UUAGGAAGUACCUCUAUGC-3′) and TLR2 (forward 5′-GCGG AAUCAACACAAUAGA-3′, reverse 5′-UCUAUUGUGUUGAUUCCGC-3′) were from MDBio Inc (Taipei, Taiwan). Dimethyl sulfoxide (DMSO), enzymes, TRIZOL, 2,3-bis-(2-methoxy-4-nitro-5-sulfophenyl)-2H-tetrazolium-5-carboxanilide (XTT) assay kit, Pyk2 siRNA (SASI_Rn01_00044067), and other chemicals were from Sigma (St. Louis, MO, USA). The specificities of pharmacological inhibitors and their functions are listed in Table 1.

### 4.2. Cell Culture and Treatment

RBA-1 cells originated from primary cultured astrocytes of neonatal rat cerebrum were naturally developed through successive cell passages [53] and used throughout this study. The purity of primary cultured astrocytes was assessed using an astrocyte-specific marker, anti-glial fibrillary acidic protein (GFAP) antibody, showing over 95% GFAP-positive astrocytes. Experiments were performed with cells from passages 4 to 35. The cytotoxicity of LPS and pharmacological inhibitors at the incubation time were checked using an XTT assay kit, showing no significant effect on cell viability. Cells were plated onto 6-well (2 ml/well) or 12-well (1 mL/well) culture plates and made quiescent at confluence by incubation in serum-free DMEM/F-12 for 24 h, and then incubated with LPS at 37 °C for the indicated time intervals. When the inhibitors were used, cells were pretreated with the individual inhibitor for 1 h before exposure to LPS.

### 4.3. Protein Preparation and Western Blotting

Cells were washed with ice-cold PBS and harvested in SDS-loading buffer (0.1 M Tris-HCl of pH 6.8, 1% SDS, 5% glycerol, 2.5% β-mercaptoethanol, and 0.02% bromophenol blue) to yield whole-cell extracts. Proteins were separated by SDS-PAGE and transferred onto Hybond-C membranes. Membranes were incubated with primary antibodies diluted at 1:1000 in Tween-Tris-buffered saline (TTBS), or an anti-GAPDH antibody used as an internal control. Membranes were washed with TTBS four times for 30 min each and then incubated with 1:1500 dilution of a secondary horseradish peroxidase-conjugated antibody for 1 h. Following washing, immunoreactive bands were detected by ECL using a UVP BioSpectrum 500 Imaging System (Upland, CA, USA). Image densitometry analyses were quantified using an UN-SCAN-IT gel software (Silk Scientific Inc., Orem, UT, USA).

### 4.4. MMP geLatin Zymography

Growth-arrested cells were incubated with LPS for the indicated time intervals. After treatment, the culture media were collected and centrifuged at 1000× *g* for 10 min at 4 °C to remove the cells and debris, and then were electrophoretically separated on 10% SDS-polyacrylamide gels copolymerized with 1 mg/mL gelatin (Sigma-Aldrich, St. Louis, MS, USA ) under non-reducing conditions, as described previously [54]. The gels were washed twice in 2.5% Triton X-100 to remove SDS, and were then incubated for 72 h with a developing buffer containing 50 mM Tris, 40 mM HCl, 200 mM NaCl, 5 mM CaCl_2_, and 0.02% Brij-35 at 37 °C before staining with Coomassie Blue R-250. After incubation, the gels were stained in 30% methanol, 10% acetic acid, and 0.5% w/v Coomassie brilliant blue for 1 h, followed by being de-stained to visualize the gelatinolytic bands (MMP-2/9) on a dark blue background. Mixed human MMP-2 and MMP-9 (Chemicon, Temecula CA, USA) were used as gelatinase standards. Because cleaved MMPs are not reliably detectable, only pro-form zymogens were quantified in this study.

### 4.5. Transient Transfection with siRNA

RBA-1 cells were plated at 2 × 10^5^ cells/mL onto 6-well plates until reaching about 70% confluence. Cells were washed once with PBS, and 1 mL/well of DMEM/F-12 with 5% FBS was added before transfection. Transient transfection of siRNA was carried out using Genmute transfection reagent (SignaGen Laboratories, Rockville, MD, USA). The transfection reagent complexes were added to each well that contained siRNA with a final concentration of 50 nM, and were then incubated at 37 °C for 5 h. After transfection, cells were incubated with DMEM/F-12 with 5% FBS for an additional 8 h, and then washed twice with PBS and maintained in serum-free DMEM/F-12 for 16 h before treatment with LPS.

### 4.6. Total RNA Extraction and Real-Time PCR Analysis

Total RNA was extracted from RBA-1 cells, as previously described [54]. The cDNA obtained from 0.5 μg total RNA was used as a template for PCR amplification. Oligonucleotide primers were designed on the basis of Genbank entries for rat MMP-9 and GAPDH. The following primers were used for amplification reaction:

MMP-9:

5′-AGTTTGGTGTCGCGGAGCAC-3′ (sense);
5′-TACATGAGCGCTTCCGGCAC-3′ (antisense);
5′-CGCTCTGCATTTCTTCAAGGACGGT-3′-tetramethylrhodamine (TAMRA, Probe)

GAPDH:

5′-(AACTTTGGCATCGTGGAAGG)-3′ (sense);
5′-(GTGGATGCAGGGATGATGTTC)-3′ (antisense);
5′- TGACCACAGTCCATGCCATCACTGC-3′-TAMRA (Probe).

Real-time PCR was performed with a TaqMan gene expression assay system, using primers and probe mixes for MMP-9 and endogenous GAPDH control genes. PCRs were performed using a 7500 Real-Time PCR System (Applied Biosystems, Foster City, CA). Relative gene expression was determined by the ΔΔCt method, where Ct is the threshold cycle. All experiments were performed in triplicate.

### 4.7. Rat MMP-9 Promoter Reporter Gene Assay

The upstream region (−1280 to +108) of the rat MMP-9 promoter was cloned into the pGL3-basic (a plasmid) vector containing the luciferase reporter system [55,56]. The plasmid was prepared by using QIAGEN plasmid DNA preparation kits. The construct was transfected into RBA-1 cells by using a Lipofectamine reagent according to the instructions of the manufacturer. The transfections were repeated at least three times to ensure reproducibility of the results. Transfection with pGal (a plasmid) encoding for β-galactosidase was used for the control of transfection efficiencies. To assess promoter activity, after incubation with LPS, cells were collected and disrupted by sonication in a lysis buffer (25 mM Tris-phosphate, pH 7.8, 2 mM ethylenediaminetetraacetic acid (EDTA), 1% Triton X-100, and 10% glycerol). After centrifugation, aliquots of the supernatants were tested for promoter activity using a luciferase assay system (Abcam, Cambridge, United Kingdom). Firefly luciferase activities were standardized for β-galactosidase activity.

### 4.8. Cell Migration Assay

RBA-1 cells were cultured to confluence in 6-well culture plates and starved with serum-free DMEM/F-12 medium for 24 h. The monolayer cells were manually scratched with a blue pipette tip to create extended and definite scratches in the center of the wells with a bright and clear field (about 2 mm). The detached cells were removed by washing once with PBS. Serum-free DMEM/F-12 medium with or without LPS (2 μg/mL) was added to each well, as indicated after pretreatment with or without individual inhibitor for 1 h. All experiments were performed under incubation with a DNA synthesis inhibitor (10 μM hydroxyurea). Images of migratory cells from the scratched boundary were observed under a light microscope with a digital camera (Olympus, Japan). Numbers of migratory cells were counted from the resulting four phase images for each point and then averaged for each experimental condition. The data presented are summarized from three separate assays.

### 4.9. Chromatin Immunoprecipitation (ChIP) Assay

To detect the association of nuclear proteins with rat MMP-9 promoter, chromatin immunoprecipitation analysis was conducted as previously described [56]. Briefly, RBA-1 cells were cross-linked with 1% formaldehyde for 10 min at 37 °C and washed thrice with ice-cold PBS containing 1 mM phenylmethylsulfonyl fluoride (PMSF) and 1% aprotinin. The cell lysates were prepared using a SDS-lysis buffer (1% SDS, 5mM ethylenediaminetetraacetic acid (EDTA), 1 mM PMSF, 50 mM Tris-HCl) and were sonicated at 4 °C until the DNA size became 200–300 base pairs. After soluble chromatin was precleared by incubation with sheared salmon sperm DNA-protein agarose A, one portion of the sample was used as a DNA input control, and the other supernatant was immunoprecipitated with an anti-c-Jun antibody and protein A beads. Following washing and elution, precipitates were heated overnight at 65 °C to reverse cross-linking of DNA and protein. DNA fragments were purified by phenol-chloroform extraction and ethanol precipitation. The purified DNA was subjected to PCR amplification using the primers specific for the region (−606 to −327, accession no. AF148065) containing the distal AP-1 binding site (−503 to −497) present in the MMP-9 promoter region, with sense primer: 5′-AGAGCCTGCTCCCAGAGGGC-3′; antisense primer: 5′-GCCAAGTCAGGCA GGACCCC-3′. PCR fragments were analyzed by 3% agarose in 1× TAE gel containing ethidium bromide, and the size (279 bp) was compared to a molecular weight marker.

### 4.10. Analysis of Data

All the data were estimated using a GraphPad Prism Program (GraphPad, San Diego, CA, USA). Data were expressed as the mean ± SEM and analyzed by one-way analysis of variance (ANOVA) followed by Tukey′s post-hoc test. *p* < 0.01 was considered significant.

## Figures and Tables

**Table 1:** Pharmacological inhibitors and their functions.

Pharmacological Inhibitor	Function
Actinomycin D	Transcription inhibitor: Binds DNA at the transcription initiation complex and prevents elongation of RNA chain by RNA polymerase.
AG1296	Inhibitor of PDGFR: Inhibits the phosphorylation of autophosphorylation sites of the PDGFR.
Akt inhibitor VIII	Inhibitor of Akt: An allosteric inhibitor of Akt1 and Akt2 that less effectively blocks Akt3 activity.
Cycloheximide	Translation inhibitor: Inhibits translation elongation through binding to the E-site of the 60S ribosomal unit and interfering with deacetylated tRNA.
c-Src inhibitor II	Inhibitor of c-Src: A potent, selective, reversible, and ATP-competitive inhibitor of Src family tyrosine kinases.
LY294002	Inhibitor of PI3K: Inhibits PI3K activity via competitive inhibition of an ATP binding site on the p85α subunit.
PF431396	Inhibitor of Pyk2: An ATP-competitive inhibitor of PYK2/FAK (focal adhesion kinase).
p38 MAPK inhibitor VIII	Inhibitor of p38 MAPK: Inhibits p38α and p38β MAP kinase.
SP600125	Inhibitor of JNK1-3: A reversible ATP-competitive inhibitor.
Tanshinone IIA	Inhibitor of AP-1: Inhibits AP-1 activity by suppressing jun-fos (members of the transcription factor AP-1 family)-DNA complex formation.
